# *Heligmosomoides neopolygyrus* Asakawa & Ohbayashi, 1986, a cryptic Asian nematode infecting the striped field mouse *Apodemus agrarius* in Central Europe

**DOI:** 10.1186/s13071-014-0457-y

**Published:** 2014-10-11

**Authors:** Grzegorz Zaleśny, Joanna Hildebrand, Anna Paziewska-Harris, Jerzy M Behnke, Philip D Harris

**Affiliations:** Department of Invertebrate Systematics and Ecology, Institute of Biology, Wrocław University of Environmental and Life Sciences, ul. Kożuchowska 5b, 51-631 Wrocław, Poland; Department of Parasitology, Institute of Genetics and Microbiology, University of Wrocław, ul. Przybyszewskiego 63, 51-148 Wrocław, Poland; KIT Biomedical Research, Royal Tropical Institute, Meibergdreef 39, 1105 AZ, Amsterdam, the Netherlands; School of Life Sciences, University of Nottingham, NG2 7RD Nottingham, UK; Natural History Museum, University of Oslo, P.O. Box 1172, N-0562 Oslo, Norway

**Keywords:** Gastro-intestinal nematode, Phylogeography, Rodent, Post-glacial expansion, Rodent, Helminth biodiversity

## Abstract

**Background:**

*Heligmosomoides polygyrus* is a widespread gastro-intestinal nematode infecting wild *Apodemus* (wood mice) throughout Europe. Using molecular and morphological evidence, we review the status of *Heligmosomoides* from *Apodemus agrarius* in Poland previously considered to be an outlying clade of *H. polygyrus*, to further resolve the status of the laboratory model species, *H. bakeri*.

**Methods:**

Morphological analysis of the male bursa and the synlophe, and molecular analyses of concatenated nuclear (28S rDNA, ITS1 and ITS2) and mitochondrial (*CO1* and *cytb*) genes, of *Heligmosomoides* collected from *Apodemus agrarius* from two sites in Poland and comparison with related heligmosomids from voles and mice in Eurasia.

**Results:**

*Heligmosomoides neopolygyrus*, a heligmosomid nematode from *Apodemus* species from China and Japan, is recognised for the first time in western Europe infecting *Apodemus agrarius* in Poland. It can be distinguished from *H. polygyrus* by the filiform externo-dorsal rays of the male copulatory bursa and the small, equally distributed longitudinal crêtes on the body. Specimens from *A. agrarius* are 20% different at ribosomal (ITS1 and ITS2) nuclear loci, and 10% different at the mitochondrial *cytb* locus from *H. polygyrus*, and in phylogenetic analyses group with the vole-infecting genus *Heligmosomum*.

**Conclusions:**

Despite morphological similarity, *H. neopolygyrus* is only distantly related to *H. polygyrus* from western European *Apodemus*, and may be more closely related to vole-infecting taxa. It was brought into Europe by the recent rapid migration of the host mice. Inclusion of *H. neopolygyrus* in phylogenies makes it clear that *Heligmosomoides* is paraphyletic, with the pika-infecting *Ohbayashinema* and the vole-infecting *Heligmosomum* nesting within it. Clarification of the European status of *H. neopolygyrus* also allows *H. bakeri*, the laboratory model species, to be seen as a terminal sister clade to *H. polygyrus*, rather than as an internal clade of the latter taxon.

**Electronic supplementary material:**

The online version of this article (doi:10.1186/s13071-014-0457-y) contains supplementary material, which is available to authorized users.

## Background

Gastro-intestinal (GI) nematodes of the genus *Heligmosomoides* are well known parasites of wild rodents, which through their strong immunosuppressive effect [1,2] may be considered architects of helminth and pathogen communities in these hosts [1]. The laboratory model, *H. bakeri* infecting *Mus*, is one of the most intensively studied GI nematodes, with detailed accounts of its immunological and molecular interactions with the host [3,4]. Nevertheless, knowledge of other species in the genus is highly confused, and even the independent specific status of *H. bakeri*, as distinct from the *Apodemus*-infecting *H. polygyrus*, is controversial and bitterly debated [5–7]. A problem with the taxonomy of *Heligmosomoides* concerns the poor standards of description and lack of type material for older species such as *H. polygyrus*, originally described as *Strongylus polygyrus* by Dujardin [8]. This problem was exacerbated by the finding that, based on comparison of a fragment of the cytochrome b (*cytb*) mitochondrial gene, *H. polygyrus* exists in its wild hosts as numerous distinct and geographically discrete clades [9–11], which may or may not represent cryptic species. Indeed, Cable *et al*. [5] concluded that the problem lay not with our understanding of the laboratory model *H. bakeri*, but with our lack of knowledge of the broader *H. polygyrus* clade. Since then, Behnke & Harris [6] have highlighted the similarity at molecular loci between *H. polygyrus corsicum*, a taxon infecting *M. musculus* on Corsica and *Apodemus* spp. in Asia Minor [10,11], and usually considered a subspecies of *H. polygyrus*, and *H. bakeri*, and it is possible that these two forms are part of the same species. If *H. polygyrus corsicum* and *H. bakeri* can be shown to form an internal clade within *H. polygyrus*, then there can be little support for considering them as distinct species. On the other hand, if these taxa represent distinct terminal clades, then the hypothesis that they are independent species would continue to deserve consideration. Scrutiny of the phylogeny of Nieberding *et al*. [10] does indeed reveal an additional *H. polygyrus* clade (their clade 4) which lies outwith the entire sampled *H. polygyrus/H. polygyrus corsicum* clade. This clade, from Russia and Poland, predominantly from the the striped field mouse *Apodemus agrarius,* was considered evidence for a northern glacial refugium for *H. polygyrus* in Central Europe [10].

This clade presents an important test of the null hypothesis that *H. bakeri* and *H. polygyrus* are part of the same taxon, and for this reason we have undertaken the present study of *Heligmosomoides* from *A. agrarius* in central Europe. The results make it clear that this clade is not identical to *H. polygyrus*, but can be linked instead to *H. neopolygyrus*, a species previously known only from Asia east of the Urals. This species not only sheds light on the evolution of *Heligmosomoides* as a whole, but also appears to represent another example of the *A. agrarius* parasitofauna imported into Europe with the natural westward migration of its host.

## Methods

### Collection of worms

*Heligmosomoides* were collected from *A. agrarius* live-trapped near Wrocław and Gdańsk in Poland. Comparative material of *H. polygyrus* was collected from *A. flavicollis* from these sites, and from *A. sylvaticus* from Norway, the UK and Ireland. *H. glareoli* was collected from bank voles (*Myodes glareolus*) from Scotland and the island of Anglesey (Wales), and representatives of *Heligmosomum mixtum* were collected from the same host from Eastern Poland (same site as described in Cable *et al*. [5]), and from Wrocław. *H. costellatum* from *Microtus arvalis* was also available from the latter site. Material of *H. polygyrus corsicum*, from the same collections used in a study by Nieberding *et al*. [10,11] was also examined. *H. bakeri* was obtained from the Nottingham laboratory colony of this species [5]. Full details of collection sites are given in Table 1. Animals were euthanised and dissected within a few hours of collection and worms found collected into 80% ethanol for long-term storage at −20 or −80°C.

**Table 1:** **Material sequenced during this study**

**Parasite**	**Host**	**Locality**	**Country**	**Latitude**	**Longitude**
*H. neopolygyrus*	*A. agrarius*	Mokry Dwór, Wrocław	Poland	51°04′57 N	17°06′13E
*H. neopolygyrus*	*A. agrarius*	Gdańsk	Poland	54°21′19 N	18°48′20E
*H. polygyrus*	*A. sylvaticus*	Jar	Norway	59°55′15 N	10°37′46E
*H. polygyrus*	*A. sylvaticus*	Weybourne Lodge Camp, Norfolk	UK	52°55′43 N	1°09′11E
*H. polygyrus*	*A. sylvaticus*	Kildare	Eire	53°09′45 N	6°55′07 W
*H. polygyrus*	*A. flavicollis*	Mokry Dwór, Wrocław	Poland	51°04′57 N	17°06′13E
*H. polygyrus corsicum*	*A. mystacinus*	Trabzon-Sumela Road	Turkey	40°50′00 N	39°42′00E
*H. glareoli*	*My. glareolus*	Moredun Institute Edinburgh	UK	55°54′53 N	3°07′26E
*H. bakeri*	*Mus musculus*	Nottingham laboratory colony			
*H. glareoli*	*My. glareolus*	Anglesey	UK	53°10′55 N	4°10′40 W
*H. mixtum*	*My. glareolus*	Urwitałt forest,	Poland	53°47′51 N	21°39′07E
*H. mixtum*	*My. glareolus*	Mokry Dwór, Wrocław	Poland	51°04′57 N	17°06′13E
*H. costellatum*	*Mi. arvalis*	Mokry Dwór, Wrocław	Poland	51°04′57 N	17°06′13E

Rodents were collected according to the legal and ethical guidelines current in the countries where they were sampled.

### Morphological methods

The synlophes of *H. polygyrus* and *H. neopolygyrus* were studied using 2 μm transverse sections of methacrylate-embedded (Sigma) females stained with 0.1% methylene blue. To determine the 3-dimensional structure of the synlophe, specimens were also stained in 0.1% silver nitrate following the method of Khrustalev and Hoberg [12], modified by developing the silver stain by exposure to bright daylight for 5 minutes. For study of the caudal bursa of males, worms were cut just anterior to the spicules and extracted in CellLytic bacterial lysis reagent (Sigma) for up to 48 h before mounting in lactophenol for photography. Phase contrast photography was performed using a Leica DM600b with Leica DC500 camera. Voucher specimens of *H. polygyrus* and *H. neopolygyrus* have been deposited in the Natural History Museum (NHM) Oslo (accession numbers NHMO C5921-C5923), NHM London (accession numbers NHMUK 2014.2.14.1, NHMUK.2014.2.14.2-7, NHMUK.2014.2.14.8-10) and the NHM of Wroclaw University. Representative DNA samples are deposited in NHM Oslo (accession numbers NHMO NEM 0001–0019).

### Molecular methods

A total of three specimens from *A. agrarius* (2 from Wrocław, one from Gdańsk) were used for molecular analysis. As the other taxa all had representative sequences within Genbank, only single specimens from each site were sequenced. DNA was extracted using the E.Z.N.A.® Tissue DNA Kit (Omega Bio-Tek, USA), and amplified using PCR specific for 3 nuclear markers (internal transcribed spacers 1 and 2 [ITS1, ITS2], and a fragment of 28S rDNA) and 2 mitochondrial markers (fragments of genes encoding cytochrome oxidase I [*CO1*] and cytochrome b [*cytb*]). Primer sequences were drawn from the literature [5,9,13–15] (for detailed information see Table 2). PCR conditions included initial denaturation in 95°C for 5 min, followed by 35 cycles: 45 s denaturation (95°C), 30 s annealing (50°C for *CO1*, 52°C for *cytb*, 54°C for 28S rDNA and 60°C for ITS1 and ITS2), 30 s elongation (72°), and a 5 min step of final elongation (72°C). PCR products were sequenced using the same primer pairs, and chromatograms inspected visually for ambiguities. Alignments were produced using ClustalX within the Mega 5.0 package [16] followed by visual inspection. Phylogenetic analysis was conducted using a Maximum Likelihood algorithm implemented in RaxML vs 8.0 [17] via the CIPRES Science Gateway portal [18]. Nuclear ribosomal analysis was conducted on concatenated sequences (1704 bp) partitioned into 28S, ITS1, 5.8S and ITS2 genes, and included *Ohbayashinema erbaevae*, previously described as a representative of a sister genus to *Heligmosomoides* and *Heligmosomum* [19,20], *H. kurilensis kobayashii* and a variety of *H. polygyrus* sequences drawn from GenBank (Table 3)*.* The concatenated alignment included sequences represented at only one or two of the 4 loci included in the alignment, but overall each nucleotide site was represented by between 66% (28S, central region of 5.8S) and 100% (central regions of ITS1 and ITS2) of the aligned sequences. After computation of the best phylogeny (100 bootstrap replicates), identical and closely similar sequences were removed iteratively to include *H. polygyrus* diversity without over-representing this taxon in the phylogeny. For *CO1* and *cytb* a concatenated alignment (1411 bp) was produced including *H. polygyrus* sequences from each of the major mitochondrial clades identified previously [9–11]. Coverage in this case included 97% of all nucleotide positions within *cytb* and 42% of all nucleotide positions within *CO1*. Eleven isolates (35% of the total), including examples of all major clades, were represented at both *cytb* and *CO1* loci. For nuclear ribosomal loci, the chosen outgroup was *Nematodirus battus*. For analysis of mitochondrial markers, *Trichostrongylus axei* was used as outgroup. To confirm the results obtained using the concatenated nuclear alignment, homology modelling of ITS2 was undertaken using the ITS2 database (http://its2.bioapps.biozentrum.uni-wuerzburg.de/; [21]) with the secondary structure for *Trichostrongylus* ITS2 originally presented by Chilton *et al*. [22] in order to optimise the alignment (see Additional file 1). This optimised ITS2 alignment was analysed alone (Additional file 1) and also incorporated into the concatenated nuclear alignment.

**Table 2:** **Primer pairs used in the study (F- forward, R- reversed)**

**Amplified gene**	**Primers (5′➔3′)**	**Amplified fragment length (bp)**	**Reference**
*CO1*	F: GGTCAACAAATCATAAAGATATTGG	559	[5]
	R: TAAACTTCAGGGTGACCAAAAAATCA		
*cytb*	F: GRAATTTTGGTAGTATRTTRG	616	[9]
	R: AGMACGYAAAATWGYAWAAGC		
ITS1	F: TTGAACCGGGTAAAGTCGT	387 – 423	[5,13]
	R: ACAACCCTGAACCAGACGTG		
ITS2	F: ACGTCTGGTTCAGGGTTGT	276 – 306	[5,14]
	R: TTAGTTTCTTTTCCTCCGCT		
28S rDNA	F: ACCCGCTGAATTTAAGCAT	619	[15]
	R: TCCGTGTTTCAAGACGG

**Table 3:** **Sequences of Heligmosomidae used in phylogenetic analysis**

**Parasite and host**	**Locality**	***CO1***	***cytb***	**ITS1**	**ITS2**	**28S rDNA**	**Source**
*H. neopolygyrus, A. agrarius*	Gdańsk, Poland	KF765455	KF765451	KF765458	KF765463	KF765468	This study
*H. neopolygyrus, A. agrarius*	Wrocław, Poland	KJ994541	KJ994551				This study
*H. polygyrus, A. flavicollis*	Wrocław, Poland	KF765456	KF765452	KF765459	KF765464	KF765469	This study
*H. polygyrus, A. sylvaticus*	Jar, Norway	KJ994543	KJ994553	KJ994557	KJ994560	Identical to KF765469	This study
*H. polygyrus, A. sylvaticus*	Kildare, Eire	KJ994542	KJ994548				This study
*H.p. polygyrus, A.sylvaticus*	Norfolk	KJ994544	KJ994549	KJ994555	KJ994558	Identical to KF765469	This study
*H. p.polygyrus, A. sylvaticus*	98911It, Italy			AM409071	AM409087	ND	9
*H. p.polygyrus, A. sylvaticus*	Slovakia 11112Sa	ND	AM408297				10
*H.p. polygyrus, A. sylvaticus*	France 10192 F	ND	AM408288				10
*H.p.polygyrus, A.sylvaticus*	Pancas, Portugal	KJ994545	KJ994550				This study
*H. p.polygyrus, A. sylvaticus*	Minorca 7672Mi	ND	AJ971171				11
*H. bakeri, M. musculus*	Nottingham strain	DQ408627	KJ994554	DQ408624	DQ408624	AM039747	5
*H. bakeri, M. musculus*	Maizels genome project ^3^	Contig 252200	Contig 252200				
*H.p. corsicum, A. mystacinus* ^1^	Turkey			AM409074	AM409090	ND	11
*H.p. corsicum, A. mystacinus* ^1^	Turkey	KJ994540	KJ994547	KJ994556	KJ994559	KJ994539	This study
*H.p. corsicum, M. musculus* ^*1*^	Corsica 9702 F3	ND	AJ971230				11
*H. glareoli, M. glareolus*	Edinburgh, Scotland UK	KF765457	KF765453	KF765460	KF765465	KF765470	This study
*H. glareoli, M. glareolus*	Urwitałt Forest, Poland	DQ408634	ND				5
*H. glareoli, M. glareolus*	Anglesey, Wales, UK	KJ994546	KJ94552				This study
*H. kurilensis kobayashii, A. speciosus*	Japan	ND	AJ971146	AM409077	AM409093	ND	10
*H. mixtum, M. glareolus*	Wrocław, Poland	ND	KF765454	KF765461	KF765466	KF765471	This study
*H. mixtum, M. glareolus*	Mazury, Poland	DQ408635	ND				5
*H. mixtum, M. glareolus*	Spain 1340	ND	AJ971145				11
*H.costellatum, M. arvalis*	Wrocław, Poland			KF765462	KF765467	KF765472	This study
*O. erbaevae, O. daurica*	Russia, Bouriatia			AY332647	AY333381	AF210038, AF210014, AF209991	20

^1^This subspecies was originally described from *Mus musculus domesticus* from Corsica; however, ITS1 and ITS2 sequences are available only for parasites collected from *Apodemus mystacinus* from Turkey and linked to specimens from Corsica by identity of their cytb sequences ([11]).

^2^Sequences for *H. p. polygyrus* Jar and *H. bakeri* cytb are truncated relative to the other isolates.

^3^Sequence obtained through *959 Nematode Genome* project (http://www.nematodes.org/nematodegenomes/index.php/959_Nematode_Genomes). ND indicates sequence not available for inclusion in the concatenated alignments (mitochondrial or nuclear). A blank cell indicates a taxon not included in the relevant alignment.

## Results

### Morphological comparison

**Figure 1:**
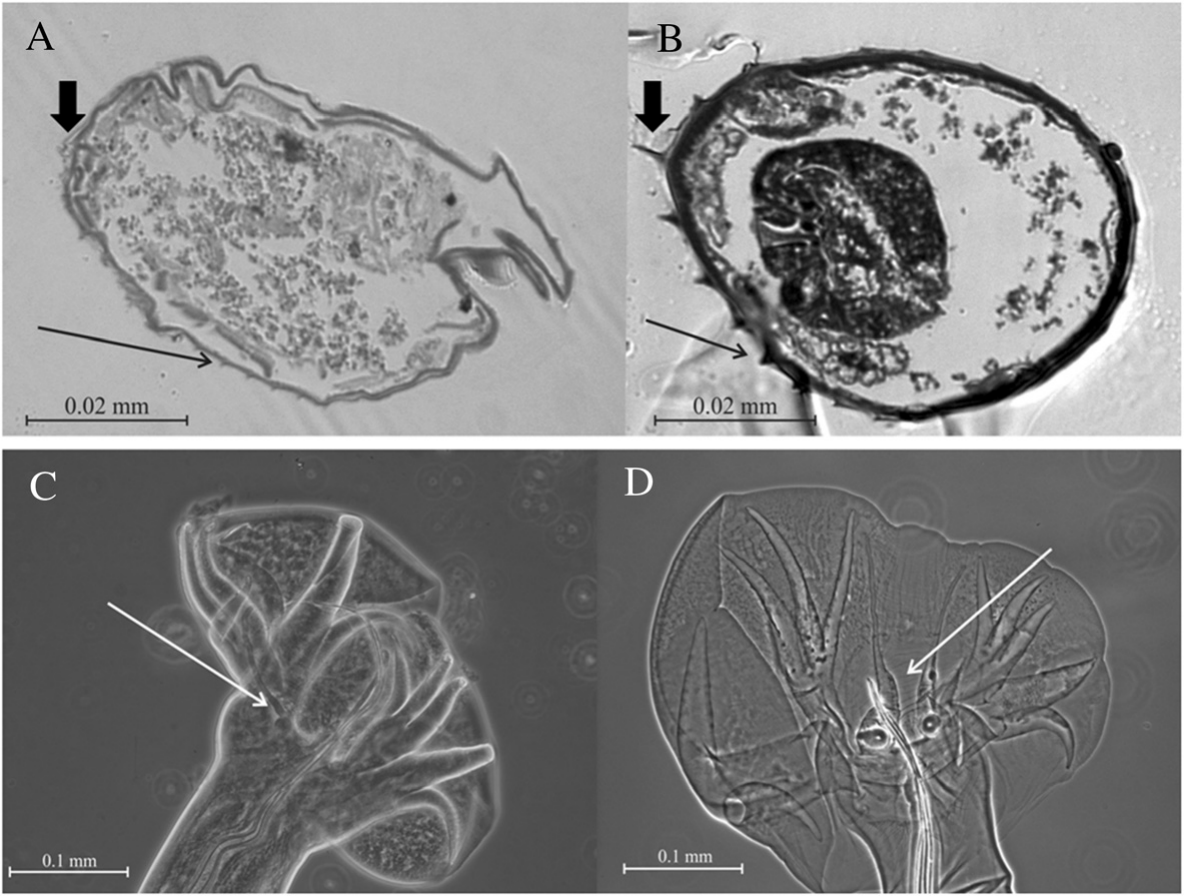
**The morphology of** ***H. polygyrus and H. neopolygyrus.*** **A)** a cross section through the mid-region of *H. polygyrus* female; **B)** a cross section through the mid-region of *H. neopolygyrus* female; In both cases, the worm is oriented with the head away from the viewer, with the dorsal surface at the top of the image. Slender black arrows indicate the ventral crête of the respective worms; the short black arrows indicate the ventral left-hand quadrant where the crête is largest in *H. polygyrus* but not in *H. neopolygyrus*. **C)** bursa of *H. neopolygyrus*; **D)** bursa of *H. polygyrus*; White arrows indicate the externo-dorsal rays, showing the enlarged base in *H. polygyrus* but the filiform ray present in *H. neopolygyrus*.

Worms from *A. agrarius* were moderately large (females up to 15 mm), reddish, slender and strongly coiled, with a habitus similar to that of *H. polygyrus*. The synlophe consisted of 18–24 longitudinal (not diagonal or partially diagonal as in *Heligmosomum*) crêtes, diagnostic for *Heligmosomoides* as re-established by Durette-Desset [23,24]. In cross-section, a clear distinction between the crêtes of the worms from *A. agrarius* and those of *H. polygyrus* was noted (Figure 1). In *H. polygyrus*, the crêtes were smallest in the dorsal right-hand quadrant of the worm and increased in size to the ventral left-hand quadrant, where the largest crête is located (Figure 1A), exactly as described by Durette-Desset [25]. The size of the crêtes increases gradually and monotonically from the smallest to the largest. In worms from *A. agrarius*, by contrast, there was no gradual increase in the size of the crêtes from dorsal right quadrant to ventral left quadrant, and instead the crêtes were more or less the same small size around the entire circumference of the worm (Figure 1B). The long filiform spicules of the worms from *A. agrarius* were similar to those from *H. polygyrus* and *H. bakeri*, with a total length of c. 700 μm. The copulatory bursa of the male worms from *A. agrarius* was asymmetrical, with the right lobe c. 30% larger than the left lobe. The arrangement of the rays was similar to that of *H. polygyrus*, with one notable difference; the externo-dorsal rays of the bursa in these worms were filiform (Figure 1C), and lacked the swollen base characteristic of *H. polygyrus* (Figure 1D).

### Phylogenetic analysis

Where comparison with accessions in Genbank was possible, sequences from *H. polygyrus*, *H. glareoli* and *H. bakeri* were similar or identical to existing curated sequences and merely extended geographical coverage, and in some cases the number of loci sequenced. Material from *Myodes glareolus* from Wrocław identified morphologically as *H. mixtum* was identical with sequences in Genbank from *M. glareolus* in NE Poland (collected and sequenced by Cable *et al.* [5] ) and with a sequence from Genbank (AJ971145) labelled ‘*Heligmosomoides costellatum*’ (sic) and deposited by Nieberding *et al*. [10]. Our worms were identified morphologically according to the criteria outlined in Tenora *et al*. [26], and moreover the Spanish *H. costellatum* sequence is recorded as collected from *Myodes glareolus*, the nominal host of *H. mixtum*. We consider this, therefore, to be an erroneously labelled example of *H. mixtum*, and our own *H. costellatum* sequences to be the first of this species to be deposited in Genbank.

The *Heligmosomoides* species from *A. agrarius* differed considerably at all sequenced loci from *H. polygyrus*. Across the 605 base 28S gene fragment, the worms from *A. agrarius* differed from *H. polygyrus* at 3 bases (0.5%), when the maximum difference noted (between *H. glareoli* and *H. polygyrus*) was 10 bases (1.6%). Across the combined ITS1/ITS2 alignment, the pairwise differences between the worms from *A. agrarius* and *H. polygyrus* was c. 5%, and even within the modelled 213 bp alignment of the stems in ITS2 (Additional file 1), there were 10 base pair differences (4.6%). The sequenced isolates of the worms from *A. agrarius* were identical at these nuclear loci. Across the 616 base pair *cytb* alignment, there were 27 base pair differences (4.4%), which were diagnostic of the worms from *A. agrarius* relative to *H. polygyrus* (other variable sites were also polymorphic within *H. polygyrus*), and within the 559 *cox1* alignment, there were 40 (7.1%) base changes. The differences between these worms and *H. polygyrus* are therefore substantial at all sequenced loci. A single base difference (T269G) was noted between the two isolates (from Wrocław and Gdańsk) at *cox1*, while their *cytb* sequences were identical. A group of *cytb* sequences in Genbank (AM408290, AM408307 - AM408312, all from Poland) were also almost identical to the sequences from the worms from *A. agrarius*, differing by up to 3 bases (0.5%) while a further sequence (AM408303) from worms collected from *A. uralensis* in Russia east of the Urals, differs from these sequences at 6 bases (0.9%).

**Figure 2:**
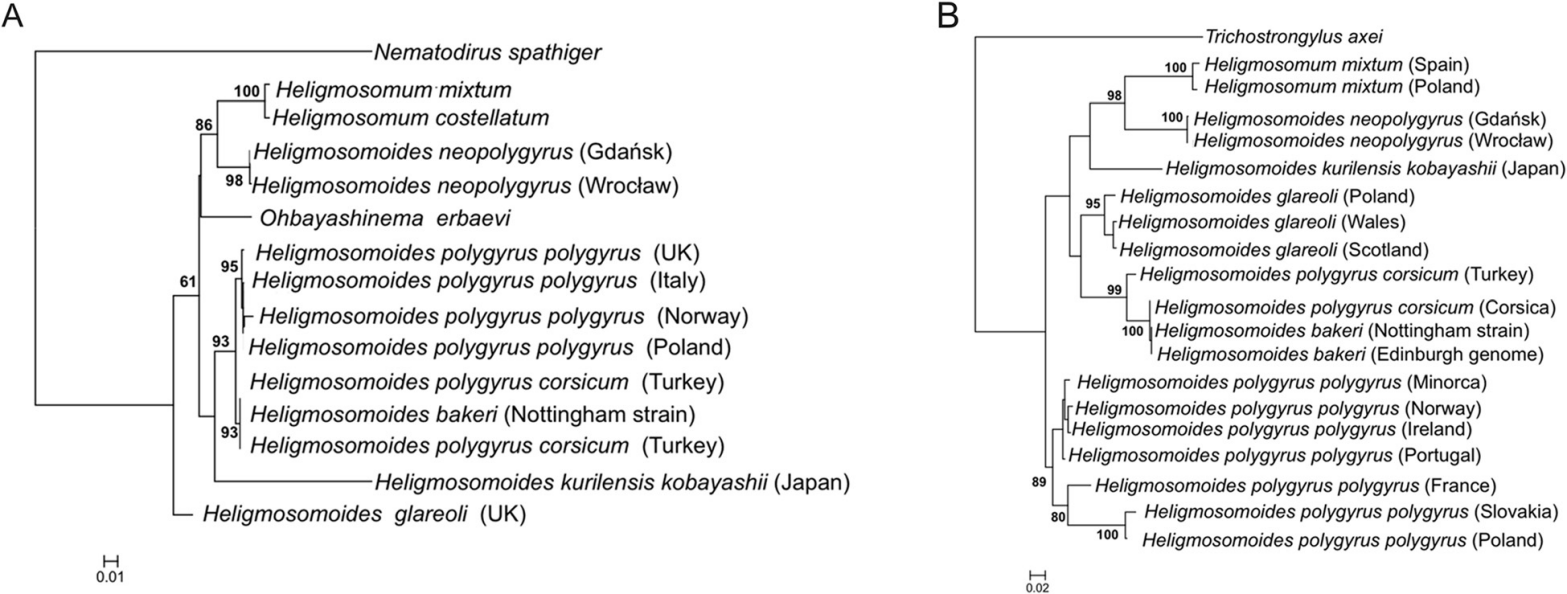
**Phylogenetic analysis of the representatives of the Heligmosomidae. A)** concatenated nuclear (28S rDNA, ITS1, 5.8S and ITS2) loci, **B)** concatenated mitochondrial loci (partial *CO1* and *cytb genes)* The analysis was performed using the Maximum Likelihood algorithm within the RaxML package implemented via the CIPRES gateway. Bootstrap support calculated on basis of 500 replicates (only values above 60% shown).

The worms from *A. agrarius* clustered consistently with the genus *Heligmosomum* at both concatenated nuclear and mitochondrial loci (Figure 2A,B). Molecular analysis of the concatenated nuclear markers (Figure 2A) recovered two strongly supported clades within the Heligmosomidae, with *Ohbayashinema erbaevi*, *H. glareoli* and *H. kurilensis kobayashii* failing to cluster with either. One of the strongly supported clades (88% bootstrap support) linked the worms from *A. agrarius* with *Heligmosomum costellatum* and *H. mixtum*. The other strongly supported clade (92% support) linked *Heligmosomoides polygyrus* with *H. polygyrus corsicum* and *H. bakeri. H. bakeri* showed higher sequence similarity to *H. polygyrus corsicum* than to *H. polygyrus polygyrus*, which formed a sister group to the *H. polygyrus corsicum*/*H. bakeri* clade. Support for these two terminal clades was 96% and 91% respectively. *H. kurilensis kobayashii* was recovered with the *H. p. polygyrus* + *H. p. corsicum*/*H. bakeri* clade, but bootstrap support for this association was weak (66%). *Ohbayashinema erbaevae* was recovered as an unresolved polytomy within the clade including *H. polygyrus*, *H. bakeri*, *H. kurilensis kobayashii* and *Heligmosomum,* while the position of *H. glareoli* was unresolved but lay outside this main *Heligmosomoides* clade. Using a more conservative alignment including a subset of 216 bases unambiguously identified as lying within base-paired stem regions of ITS2, based on homology modelling with the *Trichostrongylus/Camelostrongylus* structure of Chilton *et al*. [22] (Additional file 1), the clade including *H. polygyrus*, *H. bakeri* and *H. polygyrus corsicum*, but excluding the worms from *A. agrarius*, was recovered in 96% of bootstrap replicates, while the worms from *A. agrarius* clustered with *H. mixtum* in 70% of bootstrap replicates. No analysis revealed an association between the worms from *A. agrarius* and *H. polygyrus*.

Analysis of concatenated mitochondrial markers also failed to recover a clade grouping the worms from *A. agrarius* with *H. polygyrus* to the exclusion of other *Heligmosomoides* species (Figure 2B). Four well supported clades were recovered using the concatenated alignment, although the relative relationship of these clades to each other could not be discerned. The first well supported clade (bootstrap support 98%) included *Heligmosomum* and the worms from *Apodemus agrarius*. The other well supported groups were *H. glareoli* (bootstrap support 95%), *H. polygyrus corsicum*/*H. bakeri* (bootstrap support 99%) and *H. p. polygyrus* (bootstrap support 89%).

## Discussion

*Heligmosomoides* collected from *A. agrarius* in Poland appeared at first sight to belong to *H. polygyrus*, the common species recorded from *Apodemus* throughout Europe. The worms were red, slender and strongly coiled, with longitudinally orientated crêtes, typical of the genus as re-erected by Durette-Desset [23,24]. However, examination at two concatenated mitochondrial (*cytb* and *CO1*) and four concatenated nuclear (28S rDNA, ITS1, 5.8S rDNA and ITS2) loci make it clear that this worm is quite distinct to *H. polygyrus*, and cannot be regarded as the same species. Indeed, this taxon does not even form a sister clade to *H. polygyrus*, and clusters rather with the genus *Heligmosomum* (*H. mixtum* and *H. costellatum*). The *cytb* sequences obtained in the present work link this taxon unambiguously with specimens from *A. agrarius* collected by Nieberding *et al*. [10] from Poland (Lublin), previously misidentified because of lack of nuclear data as *H. polygyrus*. The *cytb* sequences of the Nieberding Polish material are also very similar to that (AM408303) of a specimen collected from *A. uralensis* in the region of Novosibirsk [10], suggesting that this taxon is widespread in eastern Europe and western Asia.

Four *Heligmosomoides* species have been described from *Apodemus* east of the Urals, and could be linked to the Nieberding Siberian *cytb* sequence, and therefore to the worms collected from *A. agrarius. H. kurilensis* was described from *A. speciosus* from the Kuril Islands but the male has a spicule of greater than 2 mm in length, compared to the 700 μm spicules for worms from *A. agrarius.* This species is not identical to the material considered in the present work, a view corroborated by inspection of sequences for this species deposited in Genbank. The second species from *Apodemus* in Japan, *H. desportesi* Chabaud, Rausch and Desset, 1963, is less well documented, but at least according to the habitus photographs presented by Asakawa and Ohbayashi [27], is a much more robust nematode, with a greater diameter which does not overlap with that of *H. polygyrus*, and with a symmetrical caudal bursa. This also does not appear to be identical to material collected in the present work, although there is some doubt because *H. desportesi* as redescribed by Asakawa and Ohbayashi [27] does not conform to the original description [28], and it has been suggested [27] that this material might be a synonym of *H. polygyrus*. No molecular sequences are available for this species. The third species from Japanese *Apodemus* seems a much stronger candidate for comparison with the worms from Polish *A. agrarius.* This is *H. neopolygyrus* [27], collected originally from *A. peninsulae* in Hokkaido. This has the same habitus as *H. polygyrus*, but the most convincing point of comparison concerns the slender, filiform externo-dorsal rays of the male bursa. This was noted as a specific character of *H. neopolygyrus*, as opposed to the basally swollen externo-dorsal rays of *H. polygyrus*, and is shared with the Polish material. The fourth species, *H. asakawae* [29] from Urumchi, in Western China, has expanded bases of the externo-dorsal rays, and was also originally described as *H. polygyrus* [30]; this appears to resemble *H. polygyrus* more closely than do the worms from Polish *A. agrarius*, but no molecular sequences are available. *H. neopolygyrus* has been documented on several occasions from the Asian mainland, as far west as Novosibirsk in Russia [31], and a detailed re-description was provided by Massoni *et al*. [32] based on material from Sichuan. In each case the filiform externo-dorsal rays have been highlighted as the specific character differentiating *H. neopolygyrus* from *H. polygyrus*. Furthermore, the original description of *H. neopolygyrus* highlights the small, flattened crêtes of this species, which do not increase in size to the ventral left-hand quadrant of the worm in the manner noted for *H. polygyrus*, but instead remain small and similar in size, a further character differentiating *H. neopolygyrus* from *H. polygyrus* and shared with the worms collected from Poland (Figure 1). The morphological description of *H. neopolygyrus* from Novosibirsk [31] is serendipitous because the *cytb* sequence for a *Heligmosomoides* isolate from *A. uralensis* in Novosibirsk (AM408303) is closely similar to those from our material, and also to those from Polish *A. agrarius* sequenced by Nieberding *et al*. [10]. Given the morphological and molecular agreement of the Polish worms with material from Novosibirsk, we identify the worms from *A. agrarius* in central Europe as *H. neopolygyrus*. The distribution of *A. agrarius* in Eurasia is discontinuous, with an eastern range centering on Eastern China and the Japanese islands, and a western range, extending from western China to central Europe. These two areas of distribution are separated by the Tibetan plateau. Should the molecular identity of *H. neopolygyrus* from Hokkaido be found to be different to that of specimens from Novosibirsk or the present material, the western form would require description as a new species; however, for the present we consider this to be unjustified, and consider these specimens to belong to *H. neopolygyrus*.

The genetic distance between *H. polygyrus* and *H. neopolygyrus* from Polish *A. agrarius* is considerable. At both ribosomal and mitochondrial loci, *H. neopolygrus* is much more closely associated with *Heligmosomum*, and it is clear from the phylogenies in Figure 2 that *Heligmosomoides* as a genus is paraphyletic, including both *Heligmosomum* and *Ohbayashinema*. Conventional wisdom based on morphology suggests a) that *H. neopolygyrus* and *H. polygyrus* are sister terminal clades [33,34]; b) that the *Apodemus*-infecting species are derived by host shifts from forms infecting voles [24,34,35]; and c) that the slender, coiled *Heligmosomoides* habitus is derived relative to the straight and stout *Heligmosomum* habitus. This latter assumption was implicit in Durette-Desset’s [23] resurrection of the genus *Heligmosomoides* Hall, 1916 to accommodate the spirally coiled forms; *Heligmosomum* Railliet and Henry, 1909 is the older genus, and transfer of the spirally coiled forms to the younger genus *Heligmosomoides* implies that these are derived. The hypothesis that forms of *Heligmosomoides* in *Apodemus* are ancestral to those in microtine voles deserves consideration as a more parsimonious explanation of observed data than the derivation of mouse-infecting forms from those associated with microtines suggested by Durette-Desset [34]. In the first place, *Apodemus* is one of the older murid genera, extending back to a mid-Miocene (10 MYA) origin [36,37]. Arvicolid rodents, on the other hand, are a more recent group, and the diversification of *Microtus* and *Myodes*, the principal arvicolid hosts of heligmosomids did not occur until probably 2–3 MYA [38,39]. The Eastern Asian subgenus *Apodemus* (e.g. *A. agrarius, A. latronum, A. peninsulae, A. draco*) had separated from the Western Asian/European subgenus *Sylvaemus* (including *A. sylvaticus*, *A. flavicollis*, *A. microps* and *A. mystacinus*) by 8MYA [36], and the Eastern Asian group had diversified by c. 6MYA to give rise to the progenitors of modern *A. agrarius* and *A. peninsulae* [36]. It would seem reasonable to hypothesise that *H. polygyrus* and *H. neopolygyrus* arose in *Sylvaemus* (Western) and *Apodemus* (Eastern) respectively, and that these *Heligmosomoides* species have been distinct for c. 8 MYA.

The western migration of *A. agrarius* which brought *H. neopolygrus* into Central Europe has been rapid. Although there are reports of fossil *A. agrarius* from southern France from 17 000 years BP [40], it is generally thought that the species was extinct in Europe following the last ice age, and that it has recolonised central Europe within the last few thousand years [41–43]. The earliest fossils in Poland date to c. 1000 years BP [42], and dating of hantavirus divergence suggests that the species acquired Saarema virus from *A. flavicollis* no more than 1000 years ago [41]. The recent importation of other pathogens into western Europe with this host has also been noted; Hildebrand *et al.* [44] record a range of unusual *Bartonella* genotypes in *A. agrarius*, including some most closely related to Far Eastern isolates. The extent of the secondary zone of contact between *H. neopolygyrus* and *H. polygyrus* is unclear. In Poland, the two species occur sympatrically, albeit separated by host identity, and to some extent by habitat preference; *H. neopolygyrus* was collected from *A. agrarius* at Wrocław where *A. flavicollis* sympatrically harboured *H. polygyrus*. A single worm sequenced by Nieberding *et al.* [10] but collected from *A. flavicollis* carried the *H. neopolygyrus cytb* sequence; some contact between *H. polygyrus* and *H. neopolygyrus* in eastern Europe may therefore be possible. Conversely, at the eastern limit of the range of *A. agrarius*, *H. polygyrus*-like worms were collected from *A. microps* [30] (now considered a synonym of *A. uralensis* [45]) in Urumchi, western China. This species seems able to act as a host for both *H. polygyrus* and *H. neopolygyrus* [10], and its role in maintaining the sympatry between the two *Heligmosomoides* species in Central Europe should be investigated further. Indeed, there are many records of *H. polygyrus* from rodents in Western Russia and the Caucasus (e.g. [46]), which should be re-evaluated in the light of the present description of *H. neopolygyrus* from central Europe. We would predict that *H. neopolygyrus* and *H. polygyrus* have been separated for sufficiently long that they cannot interbreed; however, given that *Caenorhabditis* species have been shown to produce viable hybrids [47] across genetic distances similar to those recorded in the present study between *H. neopolygyrus* and *H. polygyrus*, this prediction should be tested experimentally.

Finally, the present study casts new light in the debate over the specific status of the laboratory model, *H. bakeri*. Behnke & Harris [6] highlighted the similarity of *H. bakeri* and *H. polygyrus corsicum* at nuclear and mitochondrial loci. In that paper [6], the *H. polygyrus corsicum* sequences were based on a ‘composite worm’ and the present work has confirmed the molecular identity in one individual at multiple loci. As presented by Nieberding *et al*. [10], *H. p. corsicum* was a clade within *H. polygyrus*, despite its distinctness at both mitochondrial and nuclear loci [5,6], and despite the fact that it had previously been raised to an independent species [29]. It is clear from the present work that this inclusion of *H. polygyrus corsicum* within *H. polygyrus* was due to over-reliance on a paradigm that *Heligmosomoides* in *Apodemus* represents a single panmictic species [10], making a study of their divergence an exercise in population genetics rather than in taxonomy. The Nieberding *et al.* [10] ‘clade 4’ represents the eastern *H. neopolygyrus* rather than *H. polygyrus*, and there is no need to invoke a central European refugium for this clade; it was brought to Europe with the host mouse after the LGM. It is also clear from the present work that *H. bakeri/H. p. corsicum* is the sister group to *H. p. polygyrus*. From a phyletic perspective this does not confirm the specific status of the two forms, but it certainly rejects the null hypothesis that they form part of a single terminal clade. Further progress in establishing the separate identity of *H. bakeri* depends on a critical evaluation of the taxonomy of *Heligmosomoides* from *Apodemus* and *Mus* across the region from the Carpathians (the eastern limit of Nieberding’s detailed sampling) to western China, paying particular attention to such areas of high biodiversity as the Caucasus. Given the highly specific nature of the interference with the host immune system achieved by *H. bakeri* [3,4,48] and *H. polygyrus* [1,2], such a study might be an especially fruitful approach to identifying the factors responsible for speciation and host specificity of these important GI nematodes.

## Conclusions

1. *Heligmosomoides neopolygyrus* is recorded for the first time in Europe, arriving as a natural immigrant with *Apodemus agrarius*.
2. According to molecular criteria *H. neopolygyrus* and *H. polygyrus* are not closely related, and *H. neopolygyrus* may be more closely related to vole-infecting forms.
3. The vole-infecting genus *Heligmosomum* and the pika-infecting *Ohbayashinema* cluster within *Heligmosomoides*.
4. *H. bakeri* (the laboratory model) and *H. p. corsicum* are confirmed as the sister group to *H. polygyrus*, rather than representing an internal clade within the latter species.

## Additional file

Additional file 1:
**ITS2 modelling to improve phylogenetic alignment.**
