# Supplementary material for: Heligmosomoides neopolygyrus Asakawa & Ohbayashi, 1986, a cryptic Asian nematode infecting the striped field mouse Apodemus agrarius in Central Europe
Source: Parasit Vectors. 2014 Oct 11;7:457. doi: 10.1186/s13071-014-0457-y (PMC4198666; doi:10.1186/s13071-014-0457-y)
Supplement: Additional file 1: — ITS2 modelling to improve phylogenetic alignment. [file 13071_2014_457_MOESM1_ESM.pdf]

Additional file 1 – ITS2 modelling to improve phylogenetic alignment

The Chilton et al. (1998) model of *Trichostrongylus* and *Camelostrongylus* ITS2 was converted into dot and bracket notation (Supplementary appendix 1) and modelled using the ITS2 workbench (its2.bioapps.biozentrum.uni-wuerzburg.de). The ITS2 of *Heligmosomoides bakeri* is substantially longer than these previously modelled trichostrongyle ITS2 molecules; the HMMR algorithm for annotation of ITS2 sequences within the ITS2 workbench placed the ends of the ITS2 molecule as depicted in Supplementary Figure 1B, giving a length for the RNA fragment of 311 bases (compared to 227 bases for the *Trichostrongylus* molecule). Modelling of this fragment using RNAfold (www.bibiserv.techfak.uni-bielefeld.de) gave the structure indicated in Supplementary Figure 1A. This is comparable with the Chilton structure for *Trichostrongylus* and *Camelostrongylus* (Supplementary Figure 1A), which modelled using the same algorithm gave a minimum free energy for the structure (-58) close to that recorded by Chilton et al. (1998), but with substantially extended helices 2 and 3, following the nomenclature of Colman (2009), rather than the nomenclature of Chilton et al. (1998). The 5' region of helix 3 contains the conserved motif GATGTCAAC noted by Coleman (2007). Helix 2 contains 24 base pairs compared with the 13 base pairs present in *Trichostrongylus*, whereas Helix 4 contains 44 base pairs relative to the 34 seen in *Trichostrongylus*. Helix 1 is of similar length (7 bp) in both *Heligmosomoides bakeri* and *Trichostrongylus* spp, as is the short helix associated with helix 3 (Helix 6 according to Chilton et al., 1998), which includes 13 bp in *Trichostrongylus* and 11 bp in *H. bakeri*.

Based on these models of the *Heligmosomoides* ITS2, an alignment was produced which included only the unambiguously base-paired residues from the stems of the RNA helices, and which excluded residues placed within unpaired loop structures. This alignment included 216 bases, of which 36 were variable. Phylogenies produced using this alignment were similar to the alignments based on the whole concatenated nuclear sequences, and the clade including the two *Heligmosomum* species, and the clade including *Heligmosomoides bakeri* and *H. polygyrus*, were recovered in 80%-94% of bootstrap replicates, depending on the method of analysis. *H. neopolygyrus* and *H. glareoli* were consistently excluded from these two clades. Both *Ohbayashinema* and *Heligmosomum* were recovered as internal clades, with *Heligmosomoides kurilensis kobayashii* the most divergent taxon (Supplementary Figure 2). Outgroups from the Heligmonellidae or Trichostrongylidae were not included because of the divergent lengths of base paired regions in these families. Although this analysis utilised a smaller and more conservative alignment (the unambiguously aligned residues of ITS2, based on structural analysis of the RNA molecule), the conclusions were the same as those reported in the main paper for concatenated nuclear loci; *H. neopolygyrus* is never included in a single clade with *H. polygyrus* or *H. bakeri*, and both *Ohbayashinema* and *Heligmosomum* form internal clades within *Heligmosomoides*.

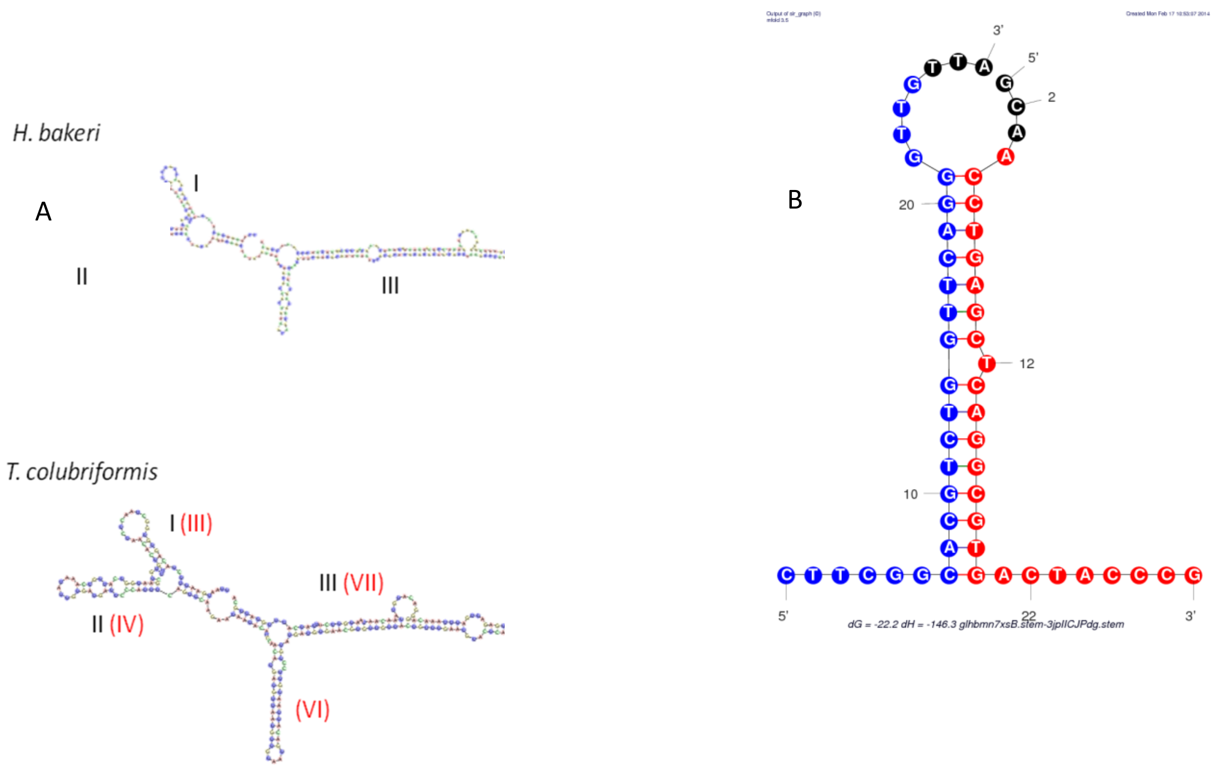

Supplementary Figure 1. A) Structures for ITS2 predicted using RNAfold for *Heligmosomoides bakeri* and *Trichostrongylus colubriformis*. Black roman numerals indicate helices as identified by Coleman (2007, 2009). Red numerals in brackets indicate the helix numbering used by Chilton et al. (1998). B) Base pairing between 5.8S RNA and 28S RNA, defining either end of ITS2, as predicted by ITS2 workbench.

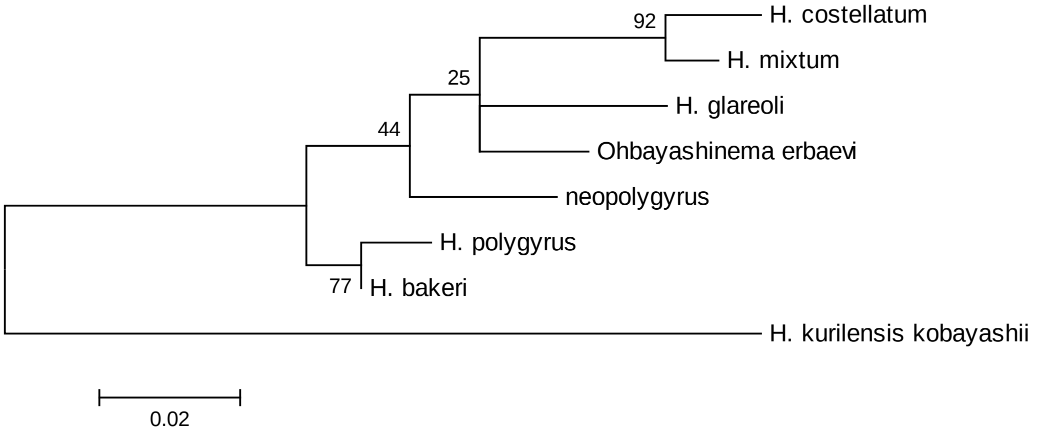

Supplementary Figure 2. Consensus tree based on 216 bases of aligned base paired regions of the ITS2 RNA of *Heligmosomoides* spp., *Heligmosomomum* and *Ohbayashinema*. Bootstrap supports derived from Maximum Likelihood algorithm.

Supplementary references

Behnke, J.M., Keymer, A.E. & Lewis, J. (1994). *Heligmosomoides polygyrus* or *Nematospiroides dubius*? *Parasitology Today* **7**, 177-179.

Cable, J., Harris, P.D., Lewis, J.W. & Behnke, J. M. (2006). Molecular evidence that *Heligmosomoides polygyrus* from laboratory mice and wood mice are separate species**.** *Parasitology* **133**,111-122.

Chilton, N.B., Hoste, H., Newton, L.A., Beveridge, I., & Gasser, R.B. (1998). Common secondary structures for the second internal transcribed spacer pre-rRNA of two subfamilies of trichostrongylid nematodes. *International Journal for Parasitology* **28,**1765-1773.

Coleman, A.W. (2007). Pan-eukaryote ITS-2 homologies revealed by RNA secondary structure. *Nucleic Acids Research* **35**, 3322-3329.

Coleman, A. W. (2009). Is there a molecular key to the level of 'biological species' in eukaryotes. A DNA guide. *Molecular Phylogenetics and Evolution* **50**, 197-203.

Gouy de Belloq, J., Ferte, H., Depaquit, J., Justine, J.-L., Tillier, A. & Durette-Desset, M.-C. (2001). Phylogeny of the Trichostrongylina (Nematoda) inferred from 28S rDNA sequences. *Molecular Phylogenetics and Evolution* **19**, 430-442.

Nieberding, C., Libois, R., Douady, C.J., Morand, S. & Michaux, J.R., (2005). Phylogeography of a nematode (*Heligmosomoides polygyrus*) in the western Palaearctic region:persistence of northern cryptic populations during ice ages? .*Molecular Ecology* **14**, 765-779

Nieberding, C.M., Durette-Desset, M.-C., Vanderpoorten, A., Casanova, J.C., Ribas, A., Deffontaine, V., Feliu, C., Morand, S., Libois, R. & Michaux, J.R. (2008). Geography and host biogeography matter for understanding the phylogeography of a parasite. Molecular Phylogenetics and Evolution **47**, 538-554.

Appendix 1. Dot and bracket notations for ITS-2 structures of *Heligmosomoides bakeri* and *Trichostrongylus colubriformis* derived from RNAfold used in this work

>readseq-8337584939465329030_tmp_116 (*Heligmosomoides bakeri*)

UUAACGAUCUACUACAGUGUGGCUUGUUUUGAACACUGUUUGUCGAAUGGCACUCGAUCACAUGUGUCG
UUGUAUCUUGUUGUACAACGUCGCCGUUGUGAUGAUUCCCAUUCUAGUGAAGAAUCGUACAGAGCAACA
UAGUGUCGUCGACGUCGUCGUCGUCACUCCUGAAUGAUGUGAACACAUUGUGUAUGUGUGUAGUGUUA
CCGUUUCGUCUAGUACUCGGCGAGAUUAUAUUGAGGAUCGAUGAAACGGGGCCGGUACGACACUACGAC
ACAGUACAUGCGUCGUUCUGGCAUCAUUUGCAUUGCA

...(((((((...(((((((..(.......).)))))))).....((((((...((.((((((.(((.((((((((......)))))))).)))...))))))))...))))))......)).))))).....((((...((((.((.
((((...)))).)).))))...((((((((((((((...(((((((((((((((((.((((((((((.....(((((..........)))))....))))))))))).........)))))))).))))).))))))..)).)))
)..)))))))))...)))). (-104.9)

>readseq-7757451773179271487_tmp_126 (*Trichostrongylus colubriformis*)

UUAAUGAAUUUCUACAGUGUGGCUAACUCUAACACUGUUUGUCGAAUGGUCAUUGUCAAAUAUUGUGA
UGAUUCCCAUUUCAGUUCAAGAAUAAUACAUGCAACAUGAUGUUAAUGUUGUAAUGACAUUAAUGUUCC
UGUAUGAUGUGAACGUGUUGUUACUGUUUGAAUGUACUCAGUGAAUUUGAGAUUGAUUUAAACAGGGA
CAUGUAUAACAAUAAUGUUCAAUUAUCAUUUGUAUUGCA

....((((((...((((((((...........))))))))).....((((((..((..(((.......)))..))..)))))).))))))....(((((((....(((.((((((((((((.....))))))))))))..))).((((((
((((((((((((((((((((((...(((((.....)))))....))))))))))).........))))))).))))))))..))))))..)))))))).. (-61.10)
